# 输血依赖型地中海贫血儿童及青少年体格发育现状调查分析

**DOI:** 10.3760/cma.j.cn121090-20240903-00333

**Published:** 2025-04

**Authors:** 哲湘 匡, 婧余 赵, 潇 于, 静 徐, 珍 高, 燕杰 刘, 安妮 王, 瑾 董, 虹 潘, 乐乐 张, 力维 方, 桂彬 吴, 昕砾 李, 均 施, 丽 徐, 文君 解

**Affiliations:** 1 中国医学科学院血液病医院（中国医学科学院血液学研究所），血液与健康全国重点实验室，国家血液系统疾病临床医学研究中心，细胞生态海河实验室，天津 300020 State Key Laboratory of Experimental Hematology, National Clinical Research Center for Blood Diseases, Haihe Laboratory of Cell Ecosystem, Institute of Hematology & Blood Diseases Hospital, Chinese Academy of Medical Sciences & Peking Union Medical College, Tianjin 300020, China; 2 天津医学健康研究院，天津 301600 Tianjin Institutes of Health Science, Tianjin 301600, China

**Keywords:** 地中海贫血, 生长迟缓, 体重消瘦, 危险因素, Thalassemia, Stunted growth, Underweight, Risk factor

## Abstract

**目的:**

调查输血依赖型地中海贫血（TDT）儿童及青少年的身高、体重发育情况，分析治疗、经济相关因素对体格发育的影响。

**方法:**

基于中国医学科学院血液病医院基因治疗TDT临床研究的专病数据库，收集2023年10月至2024年5月期间，338例TDT儿童及青少年的身高、体重发育情况，家庭经济状况及病案资料，并通过《7岁以下儿童生长标准》《7～18岁儿童青少年身高发育等级评价》及《中国居民膳食指南》对儿童及青少年年龄别身长/身高和年龄别体重指数（BMI）进行分级评价。采用Logistic回归分析家庭经济状况、疾病治疗等因素对年龄别身长/身高和年龄别BMI的影响。

**结果:**

338例患者中儿童118例、青少年220例，男192例、女146例，中位年龄12（0.8～18）岁，中位确诊年限10.3（0.5～17.9）年，其中α型、β型、αβ复合型分别为21例（6.2％）、288例（85.2％）、29例（8.6％）。患儿家庭月收入集中在3 000～5 000元（39.9％）、5 001～<10 000元（34.9％）；67.2％家庭医疗月支出<3 000元。75.5％的TDT儿童及青少年首次输血年龄在1岁以内，儿童组和青少年组输血前HGB水平≤70 g/L分别占4.2％和6.4％。青少年组输血频率<4周1次、每月输注>2 U红细胞、血清铁蛋白（SF）≥5 000 µg/L、接受去铁治疗和脾切除的比例均显著高于儿童组，差异均有统计学意义（均*P*<0.05）。338例TDT儿童及青少年中，生长发育迟缓率为26.0％，BMI消瘦率为22.8％，同时存在生长发育迟缓和BMI消瘦率为8.9％。儿童组和青少年组生长迟缓率分别为22.9％和27.7％，差异无统计学意义（*P*＝0.402）；青少年组BMI消瘦率明显高于儿童组（26.8％对15.3％，*P*＝0.023）。多因素回归显示，家庭月收入低于10 000元（5 001～<10 000元：*OR*＝5.49，95％*CI*： 1.48～35.76；3 000～5 000元：*OR*＝6.87，95％*CI*： 1.88～44.60；<3 000元：*OR*＝9.29，95％*CI*： 2.20～64.77）、输血前HGB≤70 g/L（*OR*＝3.25，95％*CI*： 1.07～10.18）、SF≥5 000 µg/L（*OR*＝3.04，95％*CI*： 1.20～7.70）均为生长迟缓的危险因素；确诊年限长为BMI消瘦的危险因素（*OR*＝1.10，95％*CI*： 1.01～1.20）。

**结论:**

SF≥5 000 µg/L、输血前HGB≤70 g/L、家庭月收入低或确诊年限长的TDT儿童及青少年更容易出现体格发育迟缓。

## Abstract (en)

**Objective:**

To investigate the physical growth status of pediatric patients with transfusion-dependent thalassemia（TDT）and analyze the effects of treatment-related and socioeconomic factors on physical growth.

**Methods:**

Based on the specialized thalassemia database from gene therapy clinical research at the Institute of Hematology & Hospital of Blood Diseases, Chinese Academy of Medical Sciences & Peking Union Medical College, we collected data on height and weight development, family economic status, and medical records of 338 pediatric patients with TDT from October 2023 to May 2024. The length/height-for-age and body mass index（BMI）-for-age were classified based on the Growth Standard for Children under 7 Years of Age, Standard for Height Level Classification among Children and Adolescents Aged 7–18 Years, and Dietary Guidelines for Chinese Residents. Logistic regression analysis was conducted to assess the effects of family economic status and disease-related treatment on length/height-for-age and BMI-for-age.

**Results:**

Among the 338 patients, 118 were children and 220 were adolescents（192 males and 146 females）, with a median age of 12 years（range: 0.8–18）and a median diagnosis duration of 10.3 years（range: 0.5–17.9）. Subtypes included α-thalassemia［21 cases（6.2％）］, β-thalassemia［288 cases（85.2％）］, and combined αβ-thalassemia［29 cases（8.6％）］. The monthly household income of patients was concentrated in 3 000–5 000 yuan（39.9％）and 5 001–10 000 yuan（34.9％）, whereas 67.2％ of the families had monthly medical expenses of <3 000 yuan. Of the patients, 75.5％ received their first transfusion before 1 year of age. The proportions of children and adolescents with pretransfusion hemoglobin（HGB）of ≤70 g/L were 4.2％ and 6.4％, respectively. Adolescents demonstrated significantly higher rates of transfusion frequency of <4 weeks/session, monthly red blood cell infusion of >2 U, serum ferritin（SF）of ≥5 000 µg/L, iron chelation therapy, and splenectomy compared with children（all *P*<0.05）. Of the 338 patients, 26.0％, 22.8％, and 8.9％ demonstrated stunted growth, underweight, and concurrent stunted growth with underweight, respectively. No significant difference was observed in the stunted growth rates between children（22.9％）and adolescents（27.7％）（*P*＝0.402）. However, the underweight rate in adolescents（26.8％）was significantly higher than that in children（15.3％）（*P*＝0.023）. The multivariate analysis determined the following risk factors for stunted growth: monthly household income of <10 000 yuan（5 001–10 000 yuan: *OR*＝5.49, 95％ *CI*: 1.48–35.76; 3 000–5 000 yuan: *OR*＝6.87, 95％ *CI*: 1.88–44.60; <3 000 yuan: *OR*＝9.29, 95％ *CI*: 2.20–64.77）, pretransfusion HGB of ≤70 g/L（*OR*＝3.25, 95％ *CI*: 1.07–10.18）, and SF of ≥5 000 µg/L（*OR*＝3.04, 95％ *CI*: 1.20–7.70）. Longer diagnostic duration was associated with underweight（*OR*＝1.10, 95％ *CI*: 1.01–1.20）.

**Conclusion:**

Children and adolescents with TDT with pretransfusion SF of ≥5 000 µg/L, HGB of ≤70 g/L, low monthly household income, or longer diagnosis duration were significantly more likely to experience delayed physical growth.

输血依赖型地中海贫血（Transfusion-dependent thalassemia, TDT）是一种严重的遗传性溶血性疾病，常于出生后3～6个月开始发病。中国目前有近30万TDT患者群体[1]，其中儿童及青少年患者面临着生长发育迟缓这一突出健康问题，包括身高体重落后、骨骼发育不良、脏器功能损害、智力发育迟缓。这些问题不仅影响患者的长期身体健康，还给家庭带来了巨大的经济负担，增加了社会的医疗资源需求[2]–[4]。开展针对TDT儿童及青少年生长发育的研究，有助于了解该群体的健康状况，改善患者生活质量，为我国政府提供精准防治和制定公共卫生政策的科学参考，进而优化医疗资源配置，减轻家庭及社会经济负担。

我们分析了338例TDT儿童及青少年的社会人口学和临床数据，旨在调查并总结TDT儿童及青少年身高体重发育现状，为后期开展大规模、多维度TDT儿童及青少年的健康评估奠定基础；并通过分析其影响因素，构建有针对性的临床干预方案，改善儿童及青少年体格，促进健康。

## 病例与方法

1. 病例资料：本研究为横断面研究，患者来源于中国医学科学院血液病医院（中国医学科学院血液学研究所）基因治疗TDT临床研究的专病数据库（临床研究伦理批件号：XY2023087-EC-2），选取2023年10月至2024年5月期间拟报名参加基因治疗临床研究的TDT患者。研究对象纳入标准：①地中海贫血诊断及分型符合文献[5]–[8]标准；②年龄≤18岁；③本人及监护人能够理解研究内容并配合研究。排除标准：①非输血依赖型；②既往接受过异基因造血干细胞移植或基因治疗；③社会人口学或病案资料缺失率大于90％。

2. 观察指标：①社会人口学特征，包括年龄、性别、身高、体重、家庭月收入、医疗费用月支出；其中，儿童及青少年分类标准参考世界卫生组织（WHO）发布的年龄分段[9]，9岁及以下为儿童，9岁以上为青少年。②临床特征，包括疾病分型、首次输血年龄、输血频率、月输血量、输血前血红蛋白（HGB）水平、目前血清铁蛋白（SF）水平、是否进行去铁治疗、是否接受过脾切除治疗。

3. 体格发育指标评估：①年龄别身长/身高：根据不同年龄段划分的身高，0～24个月称为年龄别身长，2岁以上称为年龄别身高，用以评估儿童和青少年的生长情况。儿童及青少年年龄别身长/身高的标准差评价方法：≥同性别、同年龄儿童及青少年中位身高+2个标准差（SD）为上等；<+2 SD且≥+1 SD为中上等；<+1 SD且≥−1 SD为中等；<−1 SD且≥−2 SD为中下等；<−2 SD为下等，即生长迟缓。7岁以下儿童身长/身高标准差数值参考国家卫健委发布的《7岁以下儿童生长标准》[10]，7岁及以上参考国家卫健委发布的《7～18岁儿童青少年身高发育等级评价》[11]。②年龄别体重指数（BMI）：根据不同年龄段划分，计算公式为体重（公斤）/身高^2^（平方米），用于评估儿童和青少年的体重情况；7岁以下儿童BMI分级界值点参考《7岁以下儿童生长标准》[10]，7岁及以上儿童及青少年BMI分级界值点参考《中国居民膳食指南》[12]。根据年龄别BMI将儿童及青少年分为消瘦、正常、超重、肥胖；其中，消瘦提示营养不足。

4. 样本量计算：将身高发育迟缓设为主要研究终点，根据国外文献[13]–[15]数据，该比例为25％～33％，使用以下公式计算具有人群代表性的样本量：

$$样本量＝\frac{\frac{z^{2}\times p\left(1-p\right)}{e^{2}}}{1+\left(\frac{z^{2}\times p\left(1-p\right)}{e^{2}N}\right)}$$

其中，*N*＝目标人群数量；*e*＝误差范围；*z*＝*z*分数。

取95％置信水平，选择5％的误差范围，目标人群数量为30万，由此得到样本量为288～340例。本研究最终纳入338例患者，符合样本量计算要求。

5. 抽样及调查方法：采用便利抽样法，通过报名参加基因治疗临床研究的渠道进行患者信息收集。研究护士通过微信向患者或其监护人介绍本研究并询问其意愿，在确认其知情同意后，研究护士通过微信及电话联系患者或其监护人，收集社会人口学资料和病案资料，其中病案资料需提供纸质版检查照片为证。研究护士会将收集的资料填入纸质问卷中，最终形成基因治疗临床研究的专病数据库。

6. 统计学处理：应用R 4.0.2软件进行数据处理和分析。分类变量采用例数（构成比）、连续变量采用中位数（范围）进行统计学描述。连续变量的组间比较采用独立样本*t*检验（符合正态分布）。无序分类变量采用Pearson卡方检验或Fisher精确概率法进行组间比较，有序分类变量比较采用Kruskal-Wallis秩和检验进行组间比较。采用单因素和多因素Logistic回归分析体格发育迟缓的影响因素。*P*<0.05为差异有统计学意义。

## 结果

一、一般资料

338例TDT患者男192例（56.8％）、女146例（43.2％），中位年龄12（0.8～18）岁，中位确诊年限10.3（0.5～17.9）年。α型、β型、αβ复合型分别为21例（6.2％）、288例（85.2％）、29例（8.6％），其中中间型α、中间型β、中间型β合并α、重型β、重型β合并α的患者分别为21例（6.3％）、143例（42.3％）、13例（3.8％）、145例（42.9％）和16例（4.7％）。TDT儿童及青少年家庭月收入在3 000～5 000元、5 001～<10 000元比例最高，分别为135例（40.0％）和118例（34.9％）；227例（67.1％）儿童及青少年医疗月支出<3 000元。255例（75.5％）儿童及青少年首次输血年龄在1岁以内，青少年组输血频率<4周1次为199例（90.5％），明显高于儿童组的77例（65.3％），差异有统计学意义（*χ*^2^＝30.903，*P*<0.001）。青少年中，每月输注3～4 U和>4 U红细胞的比例分别为158例（71.8％）和48例（21.8％），明显高于儿童组的40例（33.9％）和3例（2.5％），差异有统计学意义（*χ*^2^＝116.44，*P*<0.001）。儿童组输血前HGB水平>90 g/L和≤70 g/L分别为67例（56.8％）和5例（4.2％），青少年组分别为129例（58.6％）和14例（6.4％）。青少年组SF≥5 000 µg/L为27例（12.3％），高于儿童组的3例（2.5％）（*χ*^2^＝10.913，*P*<0.001）。青少年组接受去铁治疗217例（98.6％），脾切除15例（6.8％），明显高于儿童组去铁治疗104例（88.1％）和脾切除1例（0.8％），差异有统计学意义（*P*均<0.05）（表1）。305例（95％）TDT儿童及青少年口服地拉罗司进行去铁治疗（图1）。

**表1:** 338例输血依赖型地中海贫血患者社会人口学和临床特征

特征	所有患者（338例）	儿童（118例）	青少年（220例）	统计量	*P*值
性别［例（％）］				0.115	0.735
男	192（56.8）	69（58.5）	123（55.9）		
女	146（43.2）	49（41.5）	97（44.1）		
年龄［岁，*M*（范围）］	12（0.8～18）	6（0.8～9）	13（10～18）	−32.077	<0.001
确诊年限［年，*M*（范围）］	10.3（0.5～17.9）	4.9（0.5～9）	12.5（1～17.9）	−28.057	<0.001
疾病分型［例（％）］				0.214	0.643
中间型α	21（6.3）	13（11.0）	8（3.6）		
中间型β	143（42.3）	40（33.9）	103（46.8）		
中间型β合并α	13（3.8）	5（4.2）	8（3.6）		
重型β	145（42.9）	54（45.8）	91（41.5）		
重型β合并α	16（4.7）	6（5.1）	10（4.5）		
家庭月收入［例（％）］				0.214	0.643
≥10 000元	41（12.1）	13（11.0）	28（12.7）		
5 001～<10 000元	118（34.9）	38（32.2）	80（36.4）		
3 000～5 000元	135（40.0）	55（46.6）	80（36.4）		
<3 000元	44（13.0）	12（10.2）	32（14.5）		
医疗月支出［例（％）］				2.043	0.153
5 001～10 000元	12（3.6）	3（2.5）	9（4.1）		
3 000～5 000元	99（29.3）	30（25.5）	69（31.4）		
<3 000元	227（67.1）	85（72.0）	142（64.5）		
首次输血年龄［例（％）］				2.410	0.121
0～1岁	255（75.5）	94（79.7）	161（73.2）		
2～3岁	40（11.8）	15（12.7）	25（11.4）		
4～9岁	39（11.5）	9（7.6）	30（13.6）		
≥10岁	4（1.2）	0（0）	4（1.8）		
输血频率［例（％）］				30.903	<0.001
≥4周1次	62（18.3）	41（34.7）	21（9.5）		
<4周1次	276（81.7）	77（65.3）	199（90.5）		
月输血量［例（％）］				116.44	<0.001
≤2 U	89（26.3）	75（63.6）	14（6.4）		
3～4 U	198（58.6）	40（33.9）	158（71.8）		
>4 U	51（15.1）	3（2.5）	48（21.8）		
输血前HGB［例（％）］				0.050	0.823
>90 g/L	196（58.0）	67（56.8）	129（58.6）		
81～90 g/L	84（24.9）	36（30.5）	48（21.8）		
71～80 g/L	39（11.5）	10（8.5）	29（13.2）		
≤70 g/L	19（5.6）	5（4.2）	14（6.4）		
血清铁蛋白［例（％）］				10.913	<0.001
<2 000 µg/L	181（53.5）	75（63.6）	106（48.2）		
2 000～<5 000 µg/L	122（36.1）	37（31.4）	85（38.6）		
≥5 000 µg/L	30（8.9）	3（2.5）	27（12.3）		
缺失	5（1.5）	3（2.5）	2（0.9）		
去铁治疗［例（％）］				Fisher	<0.001
是	321（95.0）	104（88.1）	217（98.6）		
否	17（5.0）	14（11.9）	3（1.4）		
脾切除［例（％）］				Fisher	0.013
是	16（4.7）	1（0.8）	15（6.8）		
否	322（95.3）	117（99.2）	205（93.2）		
生长迟缓［例（％）］				0.702	0.402
是	88（26.0）	27（22.9）	61（27.7）		
否	250（74.0）	91（77.1）	159（72.3）		
BMI消瘦［例（％）］				5.200	0.023
是	77（22.8）	18（15.3）	59（26.8）		
否	261（77.2）	100（84.7）	161（73.2）		

**图1:**
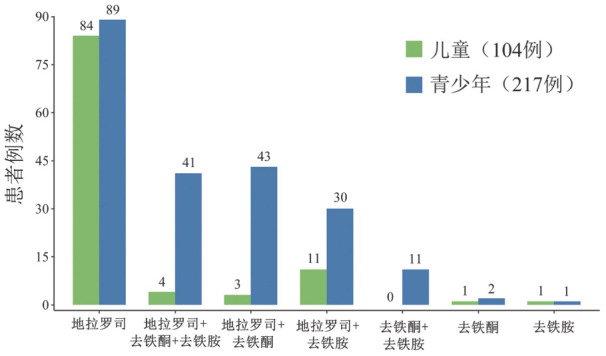
输血依赖型地中海贫血儿童及青少年去铁治疗情况

二、体格发育情况

儿童及青少年年龄别身长/身高为下等水平（即生长迟缓）88例（26.0％），中下等、中等及以上水平分别为95例（28.1％）、147例（43.5％）和8例（2.4％）；年龄别BMI为消瘦77例（22.8％）、正常241例（71.3％）、超重/肥胖20例（5.9％）（图2）。338例TDT儿童及青少年中，仅生长发育迟缓58例（17.2％），仅BMI消瘦47例（13.9％），同时存在生长发育迟缓和BMI消瘦为30例（8.9％），无生长发育迟缓和BMI消瘦为203例（60.1％）。儿童组和青少年组生长迟缓分别为27例（22.9％）和61例（27.7％），差异无统计学意义（*χ*^2^＝0.702，*P*＝0.402）；青少年组BMI消瘦59例（26.8％），明显高于儿童组的18例（15.3％）（*χ*^2^＝5.200，*P*＝0.023）（表1）。

**图2:**
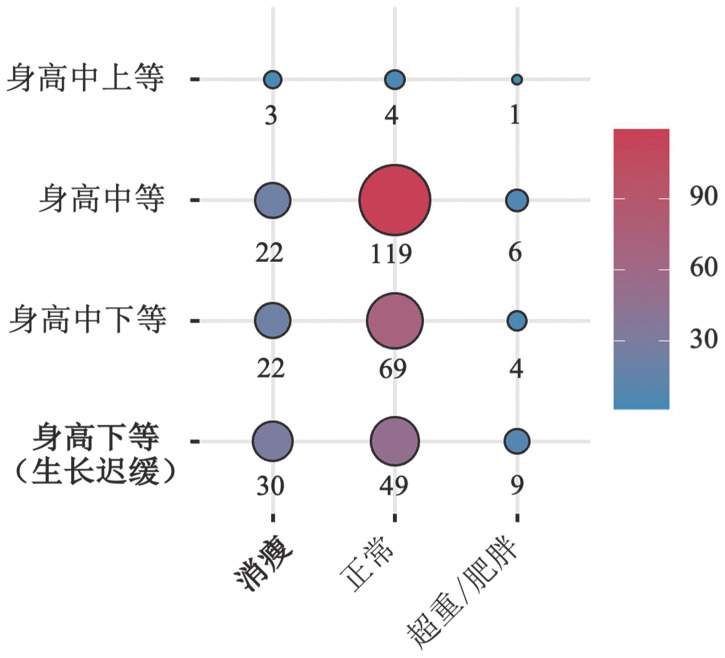
输血依赖型地中海贫血儿童及青少年年龄别身高和年龄别体重指数（BMI）分级

三、体格发育迟缓影响因素分析

为确定与TDT儿童及青少年体格发育迟缓相关的患者特征，结局事件分别定义为生长迟缓或BMI消瘦，自变量包括疾病分型、确诊年限、家庭月收入、医疗费用月支出、首次输血年龄、输血频率、月输血量、输血前HGB水平、目前SF水平、是否接受过去铁药物治疗、是否接受过脾切除治疗。

1. 生长迟缓：单因素分析显示，与生长迟缓相关的因素包括：家庭月收入水平降低、输血前HGB≤70 g/L、SF水平升高。多因素分析结果显示，家庭月收入低于10 000元（5 001～<10 000元：*OR*＝5.49，95％*CI*：1.48～35.76；3 000～5 000元：*OR*＝6.87，95％*CI*：1.88～44.60；<3 000元：*OR*＝9.29，95％*CI*：2.20～64.77）、输血前HGB≤70 g/L（*OR*＝3.25，95％*CI*：1.07～10.18）、SF≥5 000 µg/L（*OR*＝3.04，95％*CI*：1.20～7.70）均为生长迟缓的危险因素（表2）。

**表2:** 输血依赖型地中海贫血儿童及青少年生长迟缓和BMI消瘦的单因素和多因素分析

因素	生长迟缓				消瘦			
	单因素分析		多因素分析		单因素分析		多因素分析	
	*OR*（95％ *CI*）	*P*值	*OR*（95％ *CI*）	*P*值	*OR*（95％ *CI*）	*P*值	*OR*（95％ *CI*）	*P*值
疾病分型								
中间型α	Ref		Ref		Ref		Ref	
中间型β	1.43（0.49～5.22）	0.543	1.51（0.40～6.98）	0.566	0.55（0.21～1.57）	0.242	0.53（0.15～1.90）	0.313
中间型β合并α	0.77（0.10～4.69）	0.786	0.87（0.09～6.60）	0.895	0.17（0.01～1.13）	0.116	0.20（0.01～1.65）	0.190
重型β	1.73（0.60～6.29）	0.347	1.56（0.41～7.14）	0.531	0.61（0.23～1.73）	0.330	0.55（0.16～1.99）	0.348
重型β合并α	1.42（0.28～7.12）	0.664	1.27（0.20～8.28）	0.797	0.67（0.15～2.78）	0.584	0.75（0.13～4.08）	0.742
确诊年限	1.00（0.95～1.06）	0.973	0.98（0.90～1.06）	0.588	1.07（1.01～1.14）	0.025	1.10（1.01～1.20）	0.030
家庭月收入								
≥10 000元	Ref		Ref		Ref		Ref	
5 001～<10 000元	6.07（1.71～38.73）	0.017	5.49（1.48～35.76）	0.027	1.31（0.54～3.52）	0.572	1.28（0.50～3.60）	0.619
3 000～5 000元	8.51（2.45～53.78）	0.004	6.87（1.88～44.60）	0.012	1.70（0.72～4.49）	0.248	1.70（0.68～4.78）	0.280
<3 000元	12.28（3.17～81.54）	0.001	9.29（2.20～64.77）	0.007	1.43（0.49～4.35）	0.516	1.39（0.43～4.65）	0.581
医疗月支出								
5 001～<10 000元	Ref		Ref		Ref		Ref	
3 000～5 000元	1.69（0.41～11.48）	0.517	2.29（0.48～17.64）	0.348	0.81（0.22～3.88）	0.764	0.86（0.21～4.38）	0.839
<3 000元	1.84（0.47～12.17）	0.441	2.22（0.49～16.66）	0.357	0.91（0.26～4.23）	0.895	1.04（0.27～5.12）	0.956
首次输血年龄								
0～1岁	Ref		Ref		Ref		Ref	
2～3岁	1.50（0.71～3.04）	0.271	1.37（0.61～2.97）	0.428	0.85（0.35～1.86）	0.699	0.85（0.34～1.95）	0.711
4～9岁	1.22（0.55～2.54）	0.601	1.45（0.61～3.30）	0.384	1.17（0.52～2.48）	0.690	1.11（0.46～2.50）	0.810
≥10岁	3.11（0.37～26.39）	0.261	1.45（0.14～14.97）	0.745	1.13（0.06～9.03）	0.915	0.75（0.03～7.98）	0.824
输血频率								
≥4周1次	Ref		Ref		Ref		Ref	
<4周1次	0.92（0.50～1.74）	0.783	0.84（0.37～1.96）	0.673	1.01（0.54～2.02）	0.967	0.86（0.36～2.17）	0.741
月输血量								
≤2 U	Ref		Ref		Ref		Ref	
3～4 U	1.18（0.67～2.15）	0.570	1.06（0.47～2.47）	0.892	1.36（0.74～2.58）	0.337	1.05（0.44～2.56）	0.921
>4 U	1.23（0.55～2.68）	0.612	1.21（0.41～3.63）	0.728	1.30（0.56～2.99）	0.534	0.89（0.29～2.69）	0.831
输血前HGB								
>90 g/L	Ref		Ref		Ref		Ref	
81～90 g/L	1.34（0.74～2.40）	0.326	1.07（0.56～2.00）	0.844	1.26（0.69～2.27）	0.441	1.15（0.60～2.16）	0.667
71～80 g/L	1.58（0.72～3.33）	0.237	1.34（0.57～3.01）	0.494	0.92（0.37～2.06）	0.844	0.68（0.25～1.67）	0.420
≤70 g/L	3.95（1.50～10.56）	0.005	3.25（1.07～10.18）	0.038	0.94（0.26～2.78）	0.929	0.66（0.17～2.13）	0.512
血清铁蛋白								
<2 000 µg/L	Ref		Ref		Ref		Ref	
2 000～<5 000 µg/L	1.96（1.15～3.35）	0.014	1.70（0.95～3.05）	0.077	1.31（0.75～2.27）	0.332	1.29（0.70～2.36）	0.417
≥5 000 µg/L	3.78（1.67～8.53）	0.001	3.04（1.20～7.70）	0.019	2.33（1.02～5.28）	0.045	2.18（0.85～5.46）	0.099
接受去铁治疗	1.15（0.40～4.18）	0.809	1.21（0.30～5.70）	0.794	0.69（0.25～2.25）	0.506	0.45（0.12～1.89）	0.254
切脾	1.76（0.58～4.88）	0.290	1.50（0.43～4.93）	0.509	2.12（0.70～5.92）	0.159	1.41（0.43～4.28）	0.552

2. BMI消瘦：单因素分析显示，与BMI消瘦相关的因素包括：确诊年限（*OR*＝1.07，95％*CI*：1.01～1.14）、SF≥5 000 µg/L（*OR*＝2.33，95％*CI*：1.02～5.28）。多因素分析结果显示，疾病确诊年限长为BMI消瘦的危险因素（*OR*＝1.10，95％*CI*：1.01～1.20）（表2）。

## 讨论

本研究是一项针对TDT儿童及青少年体格发育情况的横断面研究。结果显示338例儿童及青少年中，26.0％存在生长发育迟缓，22.8％存在消瘦，8.9％同时存在生长发育迟缓和消瘦。根据WHO数据，全球5岁以下儿童中，22％（1.49亿）存在发育迟缓、6.7％（4 540万）BMI消瘦[16]。《中国居民营养与慢性病状况报告（2020年）》显示，6岁以下儿童生长发育迟缓率为4.8％，6～17岁儿童青少年生长迟缓率为1.7％[17]。TDT儿童及青少年生长发育迟缓率和BMI消瘦率均显著高于同龄儿童，生长发育水平有待改善。这反映了TDT儿童及青少年面临的严峻生长发育问题，并为临床管理提供了宝贵的参考。

目前，已有5项样本量超过200例的涉及TDT患者生长发育的横断面研究[18]–[22]，中国占1项[22]，其研究对象为231例重型地中海贫血患者，重点关注治疗模式和并发症，但该研究发表于2011年，数据亟待更新。此外，国内还开展了几项小样本研究[23]–[24]，缺乏大规模、系统性的流行病学调查。TDT儿童及青少年的生长发育与输血频率、铁过载及内分泌功能障碍等因素密切相关。本研究中，我们发现输血频率及输血量并非影响因素，而输血前HGB≤70 g/L是生长迟缓的危险因素。这可能是因为输血频率及输血量通常直接影响HGB水平，即输血频率、输血量这类指标影响体格发育的效应是通过HGB水平间接实现，其作用路径在统计模型中可能已被HGB水平完全解释，进而不再具有统计学意义。此外，本研究结果显示家庭月收入低于10 000元的儿童及青少年生长迟缓的风险较高。这可能与这些家庭在医疗、营养和日常护理方面的资源有限有关[25]。经济条件相对有限的家庭在获取医疗资源（如定期输血和去铁治疗）方面可能存在一定困难，且可能无法提供足够的营养支持，从而影响儿童及青少年的生长发育。在BMI消瘦方面，研究结果显示，确诊年限长是一个显著危险因素。长期的病程往往伴随着反复的输血治疗、铁过载问题以及与之相关的营养吸收不良[26]，这些因素可能会对体重产生累积性的负面影响。在本研究中，我们发现SF≥5 000 µg/L的重度铁过载患者在生长迟缓和BMI消瘦方面风险均增加。长期输血导致铁负荷增加，过量的铁沉积在器官中，引起内分泌功能紊乱和组织损伤，进而同时影响身高和体重[27]–[28]。

虽然从临床实践中可预见HGB水平、SF水平、确诊年限和家庭收入等因素可能影响体格发育，但本研究通过符合样本量要求的统计学分析，量化了每个因素对体格发育迟缓的风险增量，如“输血前HGB≤70 g/L”或“SF≥5 000 µg/L”的具体程度，为临床决策提供更加精准的依据，有助于临床干预优先级的确定和疾病管理。此外，中国TDT儿童及青少年生长发育研究缺乏大规模的流行病学调查和数据支持，这导致我们对TDT儿童及青少年的生长发育缺乏深入了解，本研究为后续开展大规模、多维度、多中心的TDT患儿健康评估提供了基础数据。本研究亦存在一定局限性。首先，研究仅纳入身高、体重指标，未探究性发育、内分泌、营养、骨密度指标，生长发育评估不够全面。其次，对于输血和去铁治疗模式的调查仍需进一步深入，以便优化治疗方案。本研究同《中国地中海贫血蓝皮书》[1]一样，采用了便利抽样法，可能存在一定选择偏倚，但该研究结果依然有临床参考价值，有助于了解地中海贫血儿童及青少年的体格发育现状。近年来，国家和地方政府加强了对TDT的防治工作。定期监测和维持HGB水平、加强铁负荷管理、优化营养管理、提供心理支持和家庭健康教育、建立完善院外管理体系、社会公益援助是改善TDT儿童及青少年生长发育的重要举措。未来，我们将继续针对中国TDT儿童及青少年生长发育开展系统性研究，全面评估该群体的健康状况，构建健康保障干预方案，提高TDT儿童及青少年的生活质量和长期健康预后。
